# Investigation of the regulatory effects of water and nitrogen supply on nitrogen transport and distribution in wolfberry fields

**DOI:** 10.3389/fpls.2024.1385980

**Published:** 2024-04-17

**Authors:** Rongrong Tian, Jinghai Wang, Minhua Yin, Yanlin Ma, Qiong Jia, Yanxia Kang, Guangping Qi, Yalin Gao, Yuanbo Jiang, Haiyan Li, Feng Xiao

**Affiliations:** College of Water Conservancy and Hydropower Engineering, Gansu Agricultural University, Lanzhou, China

**Keywords:** water and nitrogen regulation, soil NO_3_^−^–N, nitrogen uptake, N_2_O emission, CRITIC-entropy weights-TOPSIS model, wolfberry

## Abstract

Resource-based water shortages, uncoordinated irrigation, and fertilization are prevalent challenges in agricultural production. The scientific selection of appropriate water and fertilizer management methods is important for improving the utilization efficiency of agricultural resources and alleviating agricultural non-point source pollution. This study focused on wolfberry and compared the effects of four irrigation levels [full irrigation (W0, 75%–85% θ_f_), slight water deficit (W1, 65%–75% θ_f_), moderate water deficit (W2, 55%–65% θ_f_), and severe water deficit (W3, 45%–55% θ_f_)] and four nitrogen application levels [no nitrogen application (N0, 0 kg·ha^−1^), low nitrogen application (N1, 150 kg·ha^−1^), medium nitrogen application (N2, 300 kg·ha^−1^), and high nitrogen application (N3, 450 kg·ha^−1^)] on soil nitrate nitrogen (NO_3_^−^–N) transport, plant nitrogen allocation, and soil nitrous oxide (N_2_O) emissions during the harvest period of wolfberry. And this study used CRITIC-entropy weights-TOPSIS model to evaluate 16 water and nitrogen regulation models comprehensively. The results revealed the following: (1) The NO_3_^−^–N content of the soil decreased with increasing horizontal distance from the wolfberry. It initially decreased, then increased, and finally decreased with an increase in soil depth. The average NO_3_^−^–N content in the 0–100 cm soil layer ranged from 3.95–13.29 mg·kg^−1^, indicating that W0 > W1, W2, W3, and N3 > N2 > N1 > N0. (2) The soil NO_3_^−^–N accumulation ranged from 64.45–215.27 kg·ha^−1^ under varying water and nitrogen levels, demonstrating a decreasing trend with increasing horizontal distance. The NO_3_^−^–N accumulation at each horizontal distance increased with increasing irrigation and nitrogen application. The NO_3_^−^–N accumulation of W0N3 treatment increased by 5.55%–57.60% compared with the other treatments. (3) The total nitrogen content and nitrogen uptake in all wolfberry organs were W1 > W0 > W2 > W3, and N2 > N3 > N1 > N0. The maximum total nitrogen content and nitrogen uptake in W1N2 treatment were 3.25% and 27.82 kg·ha^−1^ in the roots, 3.30% and 57.19 kg·ha^−1^ in the stems, 3.91% and 11.88 kg·ha^−1^ in the leaves, and 2.42% and 63.56 kg·ha^−1^ in the fruits, respectively. (4) The emission flux and total emission of N_2_O increased with increasing irrigation and nitrogen application. The emission flux exhibited a transient peak (116.39–177.91 ug·m^−2^·h^−1^) after irrigation. The intensity of N_2_O emissions initially decreased and then increased with an increase in the irrigation amount. It also initially increased with increasing nitrogen application amount, then decreased, and finally increased again. The maximum emission intensity was observed under the W3N3 treatment (0.23 kg·kg^−1^). The N_2_O emission coefficients ranged from 0.17%–0.39%, in the order of W0 > W1 > W2 > W3 (except for N1) and N1 > N2 > N3. (5) Under varying water and nitrogen concentrations, N_2_O emission flux showed a positive linear correlation with soil pore water content and NO_3_^−^–N content and a negative linear correlation with soil temperature. The comprehensive evaluation revealed that a slight water deficit (65%–75% θ_f_) combined with medium nitrogen application (300 kg·ha^−1^) decreased soil NO_3_^−^–N leaching, increased nitrogen uptake, and reduced N_2_O emission. These findings can serve as a reference for improving the efficiency and reducing emissions of wolfberry in the Yellow River irrigation region of Gansu Province and in similar climate zones.

## 1 Introduction

The evolutionary relationship between crop growth and water and fertilizer management has a significant impact on agricultural production potential, the improvement of water and fertilizer utilization efficiency, and the prevention and control of non-point source pollution in agricultural. This has long-standing concern agricultural science (Ju et al., 2016). However, influenced by the traditional idea that high water and fertilizer usage leads to increased yields, farmers often tend to over-invest in these resources, ignoring the laws governing crop water and fertilizer demand. This results in the inadequate utilization of water and fertilizer resources and exacerbates serious agricultural non-point source pollution (Xing et al., 2021). This is inconsistent with China’s major strategic deployments such as the “one control, two reduction and three basic” requirements proposed in 2020, the zero growth in the use of fertilizers and pesticides and the effective utilization coefficient of irrigation water reaching 0.6 by 2030, and the “dual carbon” goal (Wang et al., 2022). Therefore, to further alleviate the contradiction between the water and fertilizer supply and demand and improve the soil environment, it is important to investigate farmland management strategies that involve “promoting fertilizer with water and transferring water with fertilizer” to promote the green and high-quality development of agricultural production.

Water and nitrogen play important roles in regulating crop growth and development, soil nitrogen leaching, and greenhouse gas emissions (Wang et al., 2018a). Water infiltration and redistribution can indirectly affect soil nutrient availability by affecting litter decomposition and element mineralization processes (Liu et al., 2006), ultimately enhancing soil fertility and plant nutrient absorption. Nitrogen addition can increase soil available nitrogen content and enhance plant nitrogen absorption. However, this can lead to residue problems in the soil, increasing the substrate concentration of soil microbial nitrification-denitrification, and resulting in higher soil N_2_O emissions (Song et al., 2013). In addition, the supply of water and nitrogen is not directly proportional to crop nitrogen absorption, soil inorganic nitrogen residues, or greenhouse gas emissions. On the one hand, if the water and nitrogen supply are lower than the crop absorption threshold, crop production potential will be restricted, and water and fertilizer utilization efficiency will be reduced (Liao et al., 2021). On the other hand, excessive water and nitrogen cause ecological and environmental problems, such as groundwater pollution, soil acidification, and nitrogen deposition (Yan et al., 2016; Chen et al., 2020). In context of soil-plant nitrogen transport, well-designed irrigation and fertilization strategies can minimize soil NO_3_^−^–N leaching and accumulation while enhancing crop nitrogen absorption (Azad et al., 2020). Water-saving and nitrogen-reduction measures significantly reduce soil NO_3_^−^–N leaching compared with high water and high nitrogen and increase plant biomass and nitrogen uptake (Cong et al., 2021; Mohkum et al., 2023). Irrigation with 120 mm of water coupled with nitrogen application of 180 kg·ha^−1^ can increase nitrogen content in the stems and leaves as well as promote nitrogen uptake by summer cotton plants, maximizing the total aboveground nitrogen uptake of summer cotton (Si et al., 2017). In terms of soil N_2_O emissions, the N_2_O emissions are significantly affected by water and nitrogen. In winter wheat-spring maize rotation, N_2_O emissions increase with increasing irrigation water and nitrogen application (Li et al., 2016). However, in the facility vegetable land, a combination of medium water (irrigation of 204.6 mm) and low nitrogen (nitrogen application of 75 kg·ha^−1^) compared with high water and high nitrogen (irrigation of 239.9 mm, nitrogen application of 525 kg·ha^−1^) can effectively mitigate reduce greenhouse effects and reduce the total amount of N_2_O emissions (Du et al., 2019). Irrigation of sugarcane fields with 80%–90% θ_f_ combined with nitrogen application of 250 kg·ha^−1^ can significantly reduce soil N_2_O emission flux (Chen et al., 2023). In summary, optimizing the allocation of water and nitrogen can enhance crop nitrogen accumulation, transport, and utilization, thereby effectively improving regional ecological conditions (Bai et al., 2018; Li et al., 2015).

The Yellow River irrigation region of Gansu Province is located in and arid to semi-arid inland area of northwest China. It is an important and comprehensive agricultural commodity production base in Gansu Province, with abundant light and heat resources and a significant temperature difference between day and night (Yang et al., 2019). However, the area is characterized by scarce precipitation, water scarcity, and severe secondary soil salinization (Zhao et al., 2019). Wolfberry (*Lycium barbarum* L.) is a deciduous shrub with a well-developed root system and strong resistance to cold. It has significant effects on windbreak and sand fixation, soil and water conservation, and the improvement of saline-alkaline land improvement (Danial et al., 2022). It is widely planted in the Yellow River irrigation area of Gansu Province. Previous studies on nitrogen transport have mostly focused on food crops (Mahdi et al., 2009; Mario et al., 2017; Wang et al., 2018b) and cash crops (Si et al., 2017; Du et al., 2019; Chen et al., 2023). However, there is a lack of research on economically important forest plants, such as wolfberry. In particular, research on the systematic comparison of nitrogen transport and distribution between soil, wolfberry, and the atmosphere under different water and nitrogen regulations is still rare. In view of this, this study aimed to (1) systematically analyze the distribution and accumulation of soil NO_3_^−^–N under different water and nitrogen supplies, the nitrogen distribution and absorption of wolfberry, and the characteristics and influencing factors of soil N_2_O emissions; (2) comprehensively evaluate different water and nitrogen treatments using the CRITIC-entropy weights-TOPSIS model; and (3) explore water-saving and nitrogen-reducing, water-fertilizer coupling, and environmentally friendly water and nitrogen management modes for wolfberry. This study provides a reference for the efficiency and emission reduction of wolfberry production in the Yellow River irrigation region of Gansu Province and similar arid climate areas.

## 2 Materials and methods

### 2.1 Description of the experimental site

**Figure 1:**
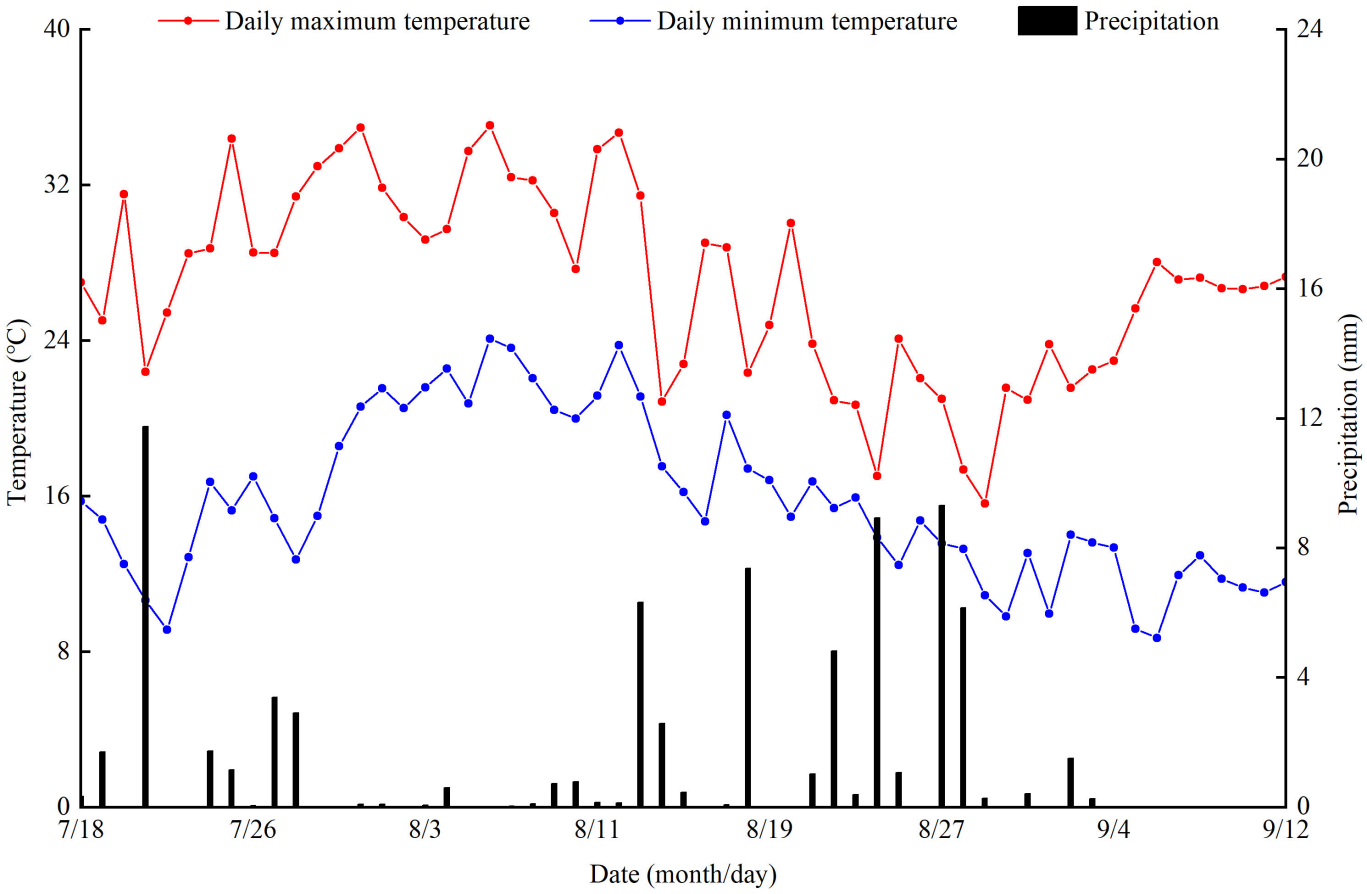
Daily distribution of precipitation and temperature during the experiment.

The experiment was conducted at the Irrigation Experimental Station (37°23′N, 104°08′E) of the Jingtaichuan Electric Power Irrigation Water Resource Utilization Center in Gansu Province from July to September 2022. This region has a temperate continental arid climate characterized by intense sunshine, infrequent rainfall, and a dry climate. The annual average sunshine duration, frost-free period, radiation amount, temperature, precipitation, and evaporation are 2652 hours, 191 days, 6.18 ×10^5^ J·cm^−2^, 8.6°C, 201.6 mm, and 3028 mm, respectively. The soil texture at the experimental site was loam, and the dry bulk density of the soil was 1.63 g·cm^−3^. The field water capacity was 24.1% (mass water content), and the pH was 8.11. Groundwater depth was > 40 m. The initial soil properties of the study site were as follows: total nitrogen 1.62 g·kg^−1^, total phosphorus 1.32 g·kg^−1^, total potassium 34.03 g·kg^−1^, available nitrogen 74.51 mg·kg^−1^, available phosphorus 26.31 mg·kg^−1^, available potassium 173 mg·kg^−1^, and alkali-hydrolyzed nitrogen 55.2 mg·kg^−1^ in the 0–60 cm soil layer. Meteorological data were collected by a small intelligent agrometeorological station installed at the experimental station. The total amount of precipitation, daily maximum temperature and daily minimum temperature during the experiment were 77.01 mm, 35.07°C and 8.71°C, respectively (**Figure 1**).

### 2.2 Experimental design and field management

**Figure 2:**
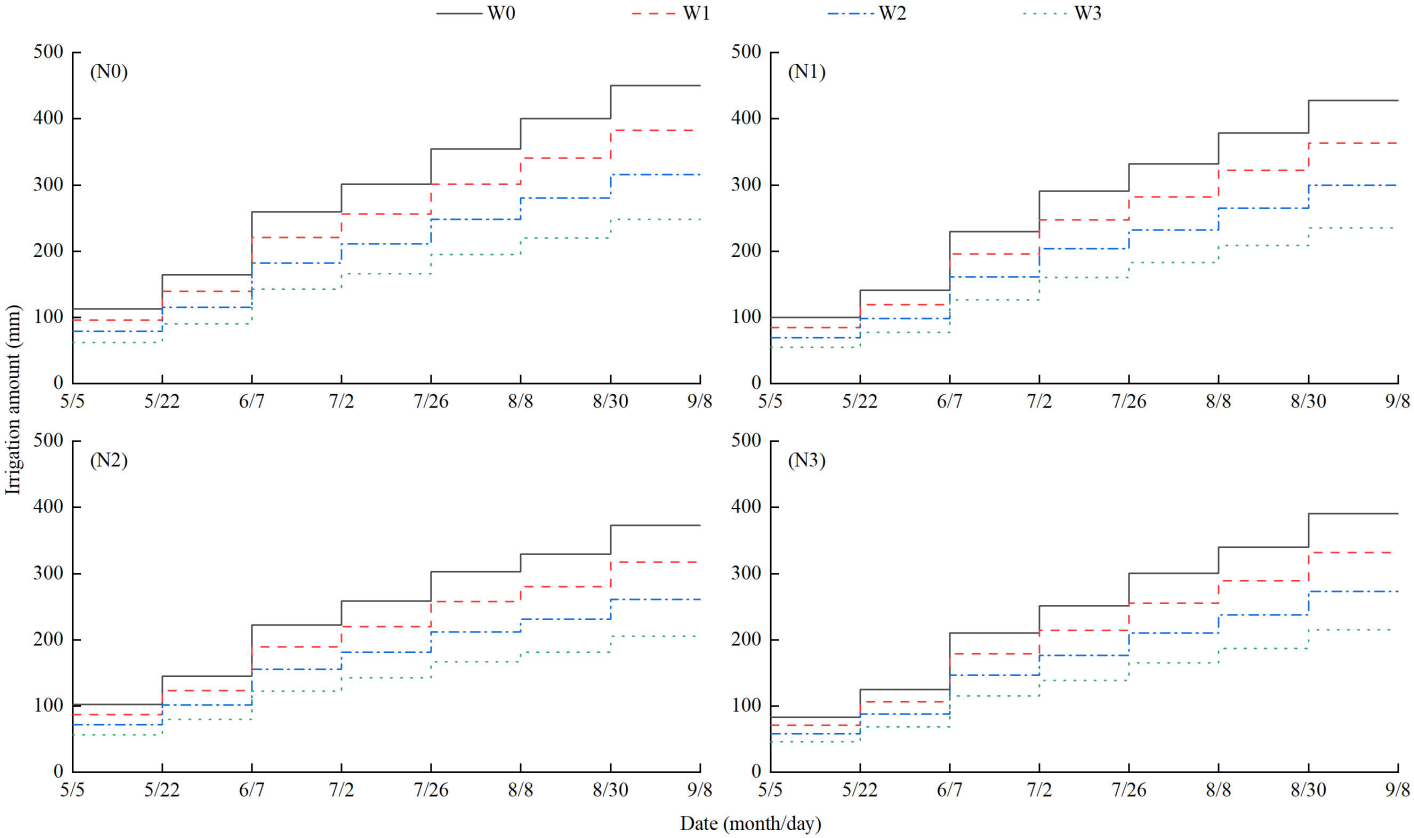
Irrigation process of each treatment during the growth of wolfberry.

The selected wolfberry (Ningqi No.5) was a two-year-old seedling transplanted on 12 April 2021, with a plant spacing of 1.5 m and row spacing of 3.0 m. Based on local production practices and previous studies (Tian et al., 2023), the experiment utilized a completely randomized block design, with irrigation and nitrogen application levels as two factors. Among them, the irrigation levels [the upper and lower limits of irrigation were set to control the percentage of soil volumetric moisture content to field water capacity (θ_f_), and the planned depth of humid layer was 60 cm] included 75%–85% θ_f_ (W0, full irrigation), 65%–75% θ_f_ (W1, slight water deficit), 55%–65% θ_f_ (W2, moderate water deficit) and 45%–55% θ_f_ (W3, severe water deficit). The nitrogen application (pure nitrogen) levels included 0 kg·ha^−1^ (N0, no nitrogen application), 150 kg·ha^−1^ (N1, low nitrogen application), 300 kg·ha^−1^ (N2, medium nitrogen application), and 450 kg·ha^−1^ (N3, high nitrogen application) (**Table 1**). Thus, there were 16 treatments in total, with each treatment repeated three times. The residential area measures 76.5 m^2^ (10.2 m × 7.5 m). Drip irrigation was then applied. Valves and water meters (with an accuracy of 0.0001 m^3^) were independently installed in the water-delivery pipes of each district to regulate the amount of irrigation effectively. The spacing of the drip irrigation belt layout was 0.3 m, the designed flow rate of the drip head was 2.0 L·h^−1^, and the spacing of the drip head was 0.3 m. The irrigation process during the wolfberry growth is illustrated in **Figure 2**. The main growth period of wolfberry in 2022 was divided into four stages: the vegetative growth period (26 April to 28 May), the full flowering period (29 May to 30 June), the full fruit period (1 July to 14 August) and the autumn fruit period (15 August to 10 September). In growing season, nitrogen fertilizer (urea and nitrogen content 46%) according to 6:2:2 was applied during the vegetative growth period (21 May), the full flowering period (7 June), and the full fruit period (4 July). Phosphate (superphosphate, with a phosphorus content of 12%) and potassium (potassium chloride, with a potassium content of 60%) at a rate of 130 kg·ha^−1^ were applied as the base fertilizer in a single application during the vegetative growth period on 21 May. Field management includes pest control and other measures consistent with those of local growers.

**Table 1:** Experimental design.

Treatment	Nitrogen application level (kg·ha^−1^)	Irrigation level (% θ_f_)	
W0N0	0	Full irrigation	75~85
W0N1	150		
W0N2	300		
W0N3	450		
W1N0	0	Slight water deficit	65~75
W1N1	150		
W1N2	300		
W1N3	450		
W2N0	0	Moderate water deficit	55~65
W2N1	150		
W2N2	300		
W2N3	450		
W3N0	0	Severe water deficit	45~55
W3N1	150		
W3N2	300		
W3N3	450		

### 2.3 Indicators and methods for measurement

#### 2.3.1 Soil nitrate-nitrogen content (NO_3_^−^–N, mg·kg^−1^)

At the end of the autumn fruit period of wolfberry, soil samples were collected using the soil drilling method. Samples were collected at 10 cm intervals from depths ranging from 0–100 cm. The collection points were located at the distances of 0.3 m, 0.6 m, 0.9 m, 1.2 m, and 1.5 m from the trunk of the wolfberry in the center of the plot. After air-drying, the soil sample was sieved through a 2 mm screen and then extracted with a 2 mol·L^−1^ KCl solution at a mass ratio of 1:10 (5 g of dry soil to liquid). The concentration of NO_3_^−^–N in the soil was subsequently measured using a UV-visible spectrophotometer (Beijing Puxi General Instrument Co., Ltd., T6 New Century) (Wang et al., 2023).

Accumulation of soil nitrate-nitrogen (*NR*, kg·ha^−1^) (Cambouris et al., 2008):


(1) $$NR=\gamma_{i}h_{i}N_{i}/10$$

where $\gamma_{i}$ is the bulk density of the soil of layer *i* (g·cm^−3^), $h_{i}$ is the soil thickness of layer *i* (cm), and $N_{i}$ is the nitrate nitrogen content of the soil in layer *i* (mg·kg^−1^).

#### 2.3.2 Total nitrogen content of wolfberry

Three representative wolfberry plants were selected from each plot for sampling during the harvest period (full fruit period and autumn fruit period). Plant samples were collected and separated into organs. They were then heated at 105 °C for 30 min, dried at 75 °C until reaching a constant weight (kg·ha^−1^), crushed, sifted through a 0.5 mm sieve, and subsequently treated with H_2_SO_4_-H_2_O_2_. The total nitrogen content of wolfberry plants was measured using the Kelley nitrogen determination method (Wu et al., 2023).

Organ nitrogen uptake of wolfberry plant (*N_u_*, kg·ha^−1^):


(2) $$N_{u}=N_{q}\times W$$

where *N_q_* represents the total nitrogen content in an organ of the wolfberry plant (%), and *W* is the dry weight of an organ of the wolfberry plant (kg·ha^−1^).

#### 2.3.3 Wolfberry yield

After ripening, wolfberries were harvested every seven days based on the plot, naturally dried, weighed, and then converted to yield per unit area (kg·ha^−1^) according to the plot area.

#### 2.3.4 Nitrous oxide emissions

Nitrous oxide gas (N_2_O) was collected and measured during the wolfberry harvest period using a closed static camera obscuru gas chromatography (Wu et al., 2022a).

#### 2.3.5 Environmental factors

(1) Soil moisture content

Each time N_2_O was collected, soil samples were collected from the topsoil layer (0–15 cm) at multiple points in each plot, and soil moisture content was determined using the drying method (105°C for 12 hours) after thorough mixing (%).

Water-filled pore water content of soil (*WFPS*, %) (Hou et al., 2016):


(3) WFPS =θ× (1 –γ/2.65) × 100%

where *θ* is the volumetric water content of the 0–15 cm soil layer (%), *γ* is the soil bulk weight (g·cm^−3^).

(2) Soil temperature

Each time N_2_O was collected, the soil temperature at a depth of 15 cm was measured next to the camera obscurum base (°C, right-angle geothermometer).

#### 2.3.6 Relevant calculation formula

(1) N_2_O emission flux (*F*, ug·m^−2^·h^−1^) (Lu et al., 2022):


(4) F=ρ×H×dc/dt×273/(273+T)×60

where *ρ* is the density of N_2_O gas in the standard state (*ρ* = 2 × 14/22.4 = 1.25) (kg·m^−3^), *H* is the height of the box (m), *dc/dt* is the rate of change of the N_2_O concentration in the box with time during the sampling process (ul·L^−1^·min^−1^), and *T* is the average temperature inside the gas collection box during the sampling process (°C).

(2) Total N_2_O emission (*f*, kg·ha^−1^) (Gao et al., 2013):


(5) $$F=\sum \left[\left(F_{i+1}+F_{i}\right)/2\;\right]\times t\times 24/10^{5}$$

where *i* is the number of samples, *t* is the number of days between the *i* sampling time and the *i+*1 sampling time (d).

(3) N_2_O emission intensity (*GHGI*, kg·kg^−1^) (Cao et al., 2022):


(6) GWP=f×298

(7) GHGI=GWP/Y

where *GWP* is the global warming potential of N_2_O (kg·ha^−1^), *f* is the total N_2_O emissions (kg·ha^−1^), and *Y* is the wolfberry yield (kg·ha^−1^).

(4) N_2_O emission coefficient (*EF*, %) (Burton et al., 2008):


(8) $$EF=\left(f_{N}–f_{0}\right)/F\times 100\%$$

where *f_N_* is the total N_2_O emissions from the nitrogen application treatment (kg·ha^−1^), *f*_0_ is the total N_2_O emissions from the treatment without nitrogen application (kg·ha^−1^), and *F* is the amount of nitrogen applied (kg·ha^−1^).

#### 2.3.7 CRITIC-entropy weights-TOPSIS model

(1) Consistency of indicator types (Ye et al., 2017; Zhou et al., 2023): Etremely large and small indicators:


(9) $$x_{ij}^{1}=\frac{1}{x_{ij}}$$

Centering indicators:


(10) $$x_{ij}^{1}=\left\{ \begin{array}{l} 2(x_{ij}-\underset{1\leq i\leq m}{\min}(x_{ij})),\underset{1\leq i\leq m}{\min}(x_{ij})\leq x_{ij}\leq \frac{\underset{1\leq i\leq m}{\max}(x_{ij})+\underset{1\leq i\leq m}{\min}(x_{ij})}{2}\\ 2(\underset{1\leq i\leq m}{\text{max}}(x_{ij})-x_{ij}),\frac{\underset{1\leq i\leq m}{\max}(x_{ij})+\underset{1\leq i\leq m}{\min}(x_{ij})}{2}\leq x_{ij}\leq \underset{1\leq i\leq m}{\max}(x_{ij}) \end{array}\right.$$

Where $x_{ij}^{1}$ is the transformed value of indicator *j* for treatment *i* to be evaluated, $\underset{1\leq i\leq m}{\min}(x_{ij})$ is the minimum value of $x_{ij}$ , $\underset{1\leq i\leq m}{\max}(x_{ij})$ is the maximum value of $x_{ij}$ , and *m* is the number of treatments to be evaluated and m=16 in this study.

(2) Data dimensionless (normalization):

In this study, the min-max standardization method was used to normalize the consistent data without dimension.


(11) $$y_{ij}=\frac{x_{ij}-\underset{1\leq i\leq m}{\min}(x_{ij})}{\underset{1\leq i\leq m}{\max}(x_{ij})-\underset{1\leq i\leq m}{\min}(x_{ij})}$$

where $y_{ij}$ is the dimensionless value of $x_{ij}$ .

(1) CRITIC-entropy weighting method to determine the weights:


(12) Qij=xij∑i=1mxij

(13) $$E_{j}=-\frac{1}{\ln(m)}\sum_{i=1}^{m}\left(Q_{ij}\times \ln Q_{ij}\right)$$

(14) $$C_{j}=S_{j}+E_{j}$$

(15) $$W_{j}=\frac{(S_{j}+E_{j})\sum_{j=1}^{n}\left(1-r_{jk}\right)}{\sum_{j=1}^{n}(S_{j}+E_{j})\sum_{j=1}^{n}\left(1-r_{jk}\right)}$$

where $Q_{ij}$ is the contribution of treatment *i* to indicator *j*, $E_{j}$ is the information entropy value of the *j* indicator, $S_{j}$ is the standard deviation of indicator *j*, $C_{j}$ is the information utility value of indicator *j*, $W_{j}$ is the weight obtained for each indicator, $r_{jk}$ is the correlation coefficient between indicator *j* and indicator *k*, and *n* is the number of evaluation indicators and n=7 in this study.

(4) Construct a weighted evaluation matrix:


(16) $$v_{ij}=y_{ij}W_{j}$$

(5) Calculate the relative closeness:


(17) $$Z^{+}=(Z_{1}^{+},Z_{2}^{+},\cdots ,Z_{n}^{+}),Z^{-}=(Z_{1}^{-},Z_{2}^{-},\cdots ,Z_{n}^{-})$$

(18) $$Z_{j}^{+}=\max(Z_{ij}),Z_{j}^{-}=\min(Z_{ij})$$

where $Z_{j}^{+}$ is the maximum value of indicator *j*, and $Z_{j}^{-}$ is the minimum value of indicator *j*.


(19) $$D_{i}^{+}=\sqrt{\sum_{j=1}^{n}{\left(z_{ij}-Z_{j}^{+}\right)}^{2}},D_{i}^{-}=\sqrt{\sum_{j=1}^{n}{\left(z_{ij}-Z_{j}^{-}\right)}^{2}}$$

(20) $$H_{i}=\frac{D_{i}^{-}}{D_{i}^{+}+D_{i}^{-}},(i=1,2,\cdots ,m)$$

where the values were sorted according to the value of $H_{i}$ . The larger values were closer to the ideal solution.

### 2.4 Data analysis

Microsoft Excel 2010 was used for data organization, Equations (1–20) were used to calculate the relevant indicators, and the CRITIC-entropy weights-TOPSIS model was used for comprehensive evaluation. IBM SPSS Statistics software (version 25.0) was used for statistical analysis. One-way ANOVA and the Duncan method were used for variance analysis and multiple comparison of indicators in different treatments (*P <* 0.05). Two-way ANOVA was performed to examine the effects of water and nitrogen, as well as their interactions, on soil NO_3_^−^–N accumulation, nitrogen uptake by wolfberry plants, and soil N_2_O emission characteristics (*P <* 0.05). The drawing was created using the Origin 2021 software.

## 3 Results

### 3.1 Soil NO_3_^−^–N distribution and accumulation under different water and nitrogen regulation

#### 3.1.1 Soil NO_3_^−^–N distribution

**Figure 3:**
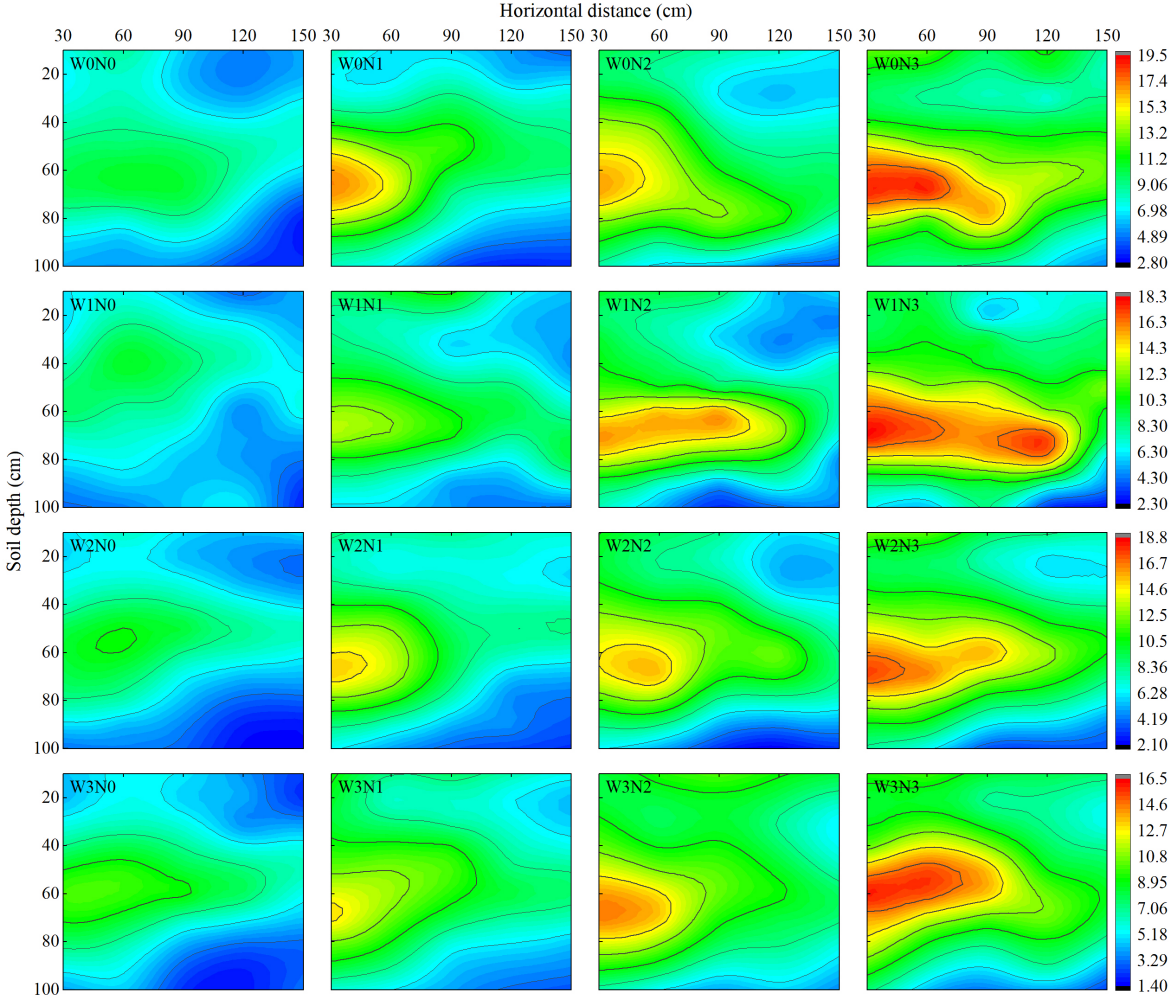
Effect of water and nitrogen regulation on NO_3_^−^–N distribution in soil. The legend on the right shows the soil NO_3_^−^–N content, unit: mg·kg^−1^. The horizontal distance represents the horizontal distance from the soil sampling point to the wolfberry plant. W0, W1, W2 and W3 refers to full irrigation (75%–85% θ_f_), slight water deficit (65%–75% θ_f_), moderate water deficit (55%–65% θ_f_) and severe water deficit (45%–55% θ_f_), respectively. N0, N1, N2 and N3 refers to the nitrogen application level is 0 kg·ha^−1^, 150 kg·ha^−1^, 300 kg·ha^−1^ and 450 kg·ha^−1^, respectively.

In the horizontal direction, the NO_3_^−^–N content of the soil decreased gradually with increasing horizontal distance from the wolfberry plant, and obvious NO_3_^−^–N accumulation zones appeared at 30 cm, 60 cm, and 90 cm from the plant. In the vertical direction, the soil NO_3_^−^–N content initially decreased, then increased, and finally decreased with increasing soil depth. An obvious NO_3_^−^–N accumulation zone appeared within the 50–80 cm soil layer (**Figure 3**). At the same irrigation level, the average NO_3_^−^–N content in soil layers 0–100 cm away was N3 (6.36–13.29 mg·kg^−1^) > N2 (5.92–12.55 mg·kg^−1^) > N1 (5.19–11.53 mg·kg^−1^) > N0 (3.95–8.39 mg·kg^−1^). The average NO_3_^−^–N contents of N1, N2, and N3 increased by 8.88%–45.06%, 15.71–66.89%, and 47.55%–78.40%, respectively, compared with N0. Under the same level of nitrogen application, at horizontal distances of 30 cm and 60 cm, the average NO_3_^−^–N content in the 0–100 cm soil layer showed that W0 > W2 > W1 and W3 as irrigation amount increased, and the average NO_3_^−^–N content of W2 decreased by 9.25% and 6.04%, respectively, compared with W0. At horizontal distances of 90 cm, 120 cm, and 150 cm, the average NO_3_^−^–N content in the 0–100 cm soil layer showed an increasing trend with increasing irrigation amount, and the average NO_3_^−^–N content of W3 decreased by 20.48%, 18.86%, and 26.25%, respectively, compared with that of W0.

#### 3.1.2 Soil NO_3_^−^–N accumulation

Irrigation and nitrogen application had extremely significant effects on the accumulation of NO_3_^−^–N in the soil layers from 0–100 cm at each horizontal distance. Their interaction effects only had extremely significant effects on NO_3_^−^–N accumulation in the soil layers from 30 cm horizontally (**Table 2**). Overall, the total NO_3_^−^–N content in the soil gradually decreased with increasing horizontal distance. The cumulative amount of NO_3_^−^–N at 150 cm was 22.57%–46.14% lower than that at 30 cm. With an increase in nitrogen application rate, the accumulation of NO_3_^−^–N at each horizontal distance significantly increased. The NO_3_^−^–N accumulation of N0, N1, and N2 decreased significantly by 32.22%–43.95%, 12.63%–32.71%, and 3.87%–29.97%, respectively, compared with N3. With an increase in the amount of irrigation, the accumulation of NO_3_^−^–N at different horizontal distances fluctuated and increased. The NO_3_^−^–N accumulation in W3 significantly decreased by 7.01%–31.79% compared with that of W0.

**Table 2:** Effects of water and nitrogen regulation on soil NO_3_^−^–N accumulation at different horizontal distances from wolfberry plants (kg·ha^−1^).

Treatment	30 cm	60 cm	90 cm	120 cm	150 cm	The average value
W0N0	130.64 ± 5.85h	136.68 ± 7.66gh	127.97 ± 9.95efg	101.82 ± 8.60efgh	91.72 ± 8.54efg	117.77 ± 7.85ef
W0N1	187.89 ± 5.73de	164.99 ± 12.28def	139.33 ± 15.36cdef	114.81 ± 7.08cdef	110.71 ± 14.15bcde	143.31 ± 10.84cd
W0N2	203.33 ± 8.32b	182.80 ± 4.51bc	154.98 ± 13.82cd	133.20 ± 10.61bc	122.45 ± 7.77ab	159.35 ± 8.92bc
W0N3	215.27 ± 4.29a	201.75 ± 10.39a	189.88 ± 9.62a	170.63 ± 11.45a	152.01 ± 13.33a	185.91 ± 9.76a
W1N0	107.74 ± 7.04i	121.97 ± 5.90hi	111.19 ± 14.80ghi	92.28 ± 6.50gh	83.42 ± 12.76fgh	103.32 ± 9.33fg
W1N1	154.84 ± 10.37g	148.40 ± 14.51fg	128.18 ± 11.25efg	109.34 ± 6.47defg	103.86 ± 11.38cdef	128.92 ± 10.56de
W1N2	179.12 ± 4.43ef	160.64 ± 6.19def	142.58 ± 11.61cde	111.48 ± 10.09bcd	96.53 ± 9.00efg	138.07 ± 8.08d
W1N3	192.21 ± 3.46cd	183.68 ± 6.47bc	177.54 ± 14.53ab	159.19 ± 10.73a	126.28 ± 12.82b	167.68 ± 9.58b
W2N0	117.51 ± 4.87i	126.08 ± 10.33hi	105.22 ± 7.72hi	84.74 ± 12.01hi	79.25 ± 13.01gh	102.56 ± 8.65fg
W2N1	170.38 ± 8.11f	154.85 ± 13.94ef	121.47 ± 7.87efgh	101.84 ± 9.62efgh	95.55 ± 11.43efg	128.82 ± 10.16de
W2N2	181.24 ± 2.56ef	174.07 ± 8.21bcd	138.70 ± 15.86cdef	116.24 ± 9.11cde	103.15 ± 11.05cdef	142.68 ± 9.32cd
W2N3	201.4 ± 3.92bc	189.76 ± 9.45ab	159.95 ± 10.85bc	136.71 ± 13.26b	118.76 ± 9.03bcd	161.32 ± 8.77b
W3N0	108.31 ± 8.87i	114.33 ± 5.56i	90.63 ± 8.42i	72.91 ± 12.43i	64.45 ± 11.76h	90.12 ± 9.33g
W3N1	157.12 ± 4.86g	135.88 ± 8.67gh	116.74 ± 13.12fgh	95.50 ± 13.35fgh	84.63 ± 7.06fg	117.97 ± 9.17ef
W3N2	171.61 ± 1.74f	156.53 ± 8.69def	136.94 ± 14.01def	123.86 ± 15.08bcd	98.96 ± 9.70defg	137.58 ± 9.83d
W3N3	179.84 ± 6.23ef	168.69 ± 12.83cde	142.45 ± 9.38cde	130.01 ± 6.05bc	103.68 ± 7.28cdef	144.94 ± 7.84cd
Test of variance of significance						
Irrigation (W)	16.484**	17.81**	14.60**	13.35**	17.05**	19.75**
Nitrogen (N)	55.781**	89.71**	51.57**	74.87**	35.81**	93.67**
W×N	3.344**	0.19ns	0.84ns	1.28ns	1.13ns	0.58ns

Different lowercase letters indicate significant differences between treatments (*P* < 0.05). W and N refer to irrigation and nitrogen application levels, respectively; N × W refers to interaction effect between the two. ** indicates an extremely significant difference (*P* < 0.01); ns indicates no significant difference (*P* > 0.05). W0, W1, W2 and W3 refers to full irrigation (75%–85% θ_f_), slight water deficit (65%–75% θ_f_), moderate water deficit (55%–65% θ_f_) and severe water deficit (45%–55% θ_f_), respectively. N0, N1, N2 and N3 refers to the nitrogen application level is 0 kg·ha^−1^, 150 kg·ha^−1^, 300 kg·ha^−1^ and 450 kg·ha^−1^, respectively.

### 3.2 Nitrogen allocation and uptake of wolfberry plants under different water and nitrogen regulation

#### 3.2.1 Nitrogen allocation

**Figure 4:**
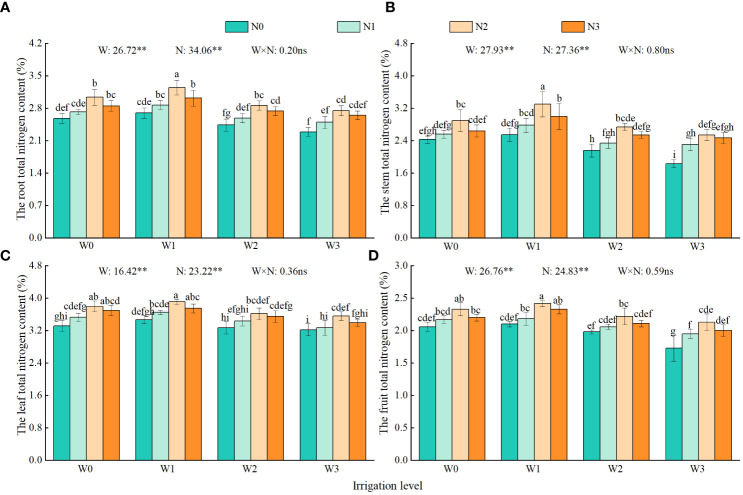
Effects of water and nitrogen regulation on total nitrogen content in each organ of wolfberry. Different lowercase letters indicate significant differences between treatments (*P <* 0.05). **(A–D)** represents the total nitrogen content of the root, stem, leaf and fruit of wolfberry, respectively. W and N refer to irrigation and nitrogen application levels, respectively; N × W refers to interaction effect between the two. ** indicates an extremely significant difference (*P <* 0.01); ns indicates no significant difference (*P* > 0.05). W0, W1, W2 and W3 refers to full irrigation (75%–85% θ_f_), slight water deficit (65%–75% θ_f_), moderate water deficit (55%–65% θ_f_) and severe water deficit (45%–55% θ_f_), respectively. N0, N1, N2 and N3 refers to the nitrogen application level is 0 kg·ha^−1^, 150 kg·ha^−1^, 300 kg·ha^−1^ and 450 kg·ha^−1^, respectively.

Irrigation and nitrogen application had a significant affect the total nitrogen content of each organ of the wolfberry, but their interaction did not have a significant effect on the total nitrogen content of each organ of the wolfberry (**Figure 4**). The total nitrogen content of each organ showed that leaves (3.22–3.91%) had higher levels than roots (2.29–3.25%), stems (1.83–3.30%), and fruits (1.73–2.42%). Under the same irrigation level, the total nitrogen content of the roots, stems, leaves, and fruits followed the order N2 > N3 > N1 > N0. The total nitrogen content of each organ in N2 increased by 17.21%–20.09%, 19.34%–38.80%, 10.56%–14.46%, and 12.12%–23.12%, respectively, compared with N0. Under the same level of nitrogen application, the total nitrogen content of roots, stems, leaves and fruits of wolfberry were in the order W1 > W0 > W2 > W3, and the total nitrogen content of each organ in W1 increased by 13.96%–18.18%, 20.35%–39.34%, 7.76%–11.62%, and 11.79%–21.39%, respectively, compared with W3.

#### 3.2.2 Nitrogen uptake

**Figure 5:**
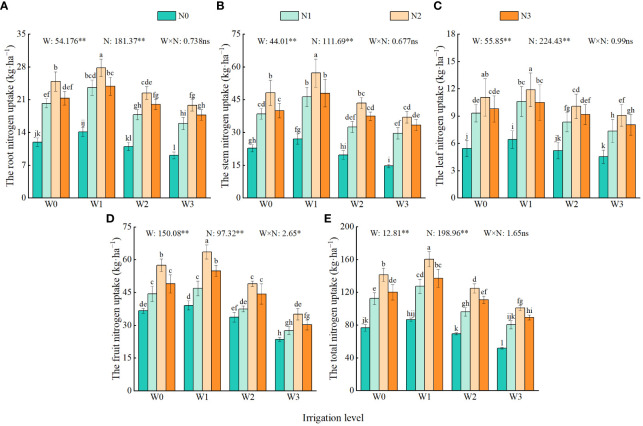
Effects of water and nitrogen regulation on nitrogen uptake of each organ of wolfberry. Different lowercase letters indicate significant differences between treatments (*P <* 0.05). **(A–E)** represents the root, stem, leaf, fruitf and total nitrogen uptake of wolfberry organs, respectively. W and N refer to irrigation and nitrogen application levels, respectively; N × W refers to interaction effect between the two. ** indicates an extremely significant difference (*P <* 0.01); * indicates a significant difference (*P* < 0.05); ns indicates no significant difference (*P* > 0.05). W0, W1, W2 and W3 refers to full irrigation (75%–85% θ_f_), slight water deficit (65%–75% θ_f_), moderate water deficit (55%–65% θ_f_) and severe water deficit (45%–55% θ_f_), respectively. N0, N1, N2 and N3 refers to the nitrogen application level is 0 kg·ha^−1^, 150 kg·ha^−1^, 300 kg·ha^−1^ and 450 kg·ha^−1^, respectively.

Irrigation and nitrogen application significantly affected the nitrogen uptake in each organ of the wolfberry. The interaction effects of these factors on nitrogen uptake varied across different organs (roots, stems, leaves, and fruits) (**Figure 5**). With an increase in irrigation and nitrogen application, the nitrogen uptake of wolfberry initially increased and then decreased. The nitrogen uptake showed that the stem and fruit had the highest uptake, followed by roots and leaves, accounting for 19.23%–87.73%, 31.04%–90.16%, 11.39%–40.80%, and 5.63%–16.78% of the total nitrogen uptake, respectively. From the perspective of total nitrogen absorption, W0, W2 and W3 decreased by 11.31%–12.50%, 19.22%–24.47%, and 34.87%–40.13%, respectively, compared with W1. N2 increased by 84.27%–94.63%, 25.51%–29.88%, and 12.70%–17.90% compared with N0, N1, and N3, respectively.

### 3.3 Soil N_2_O emission and its influencing factors under different water and nitrogen regulation

#### 3.3.1 Soil N_2_O emission parameters

**Figure 6:**
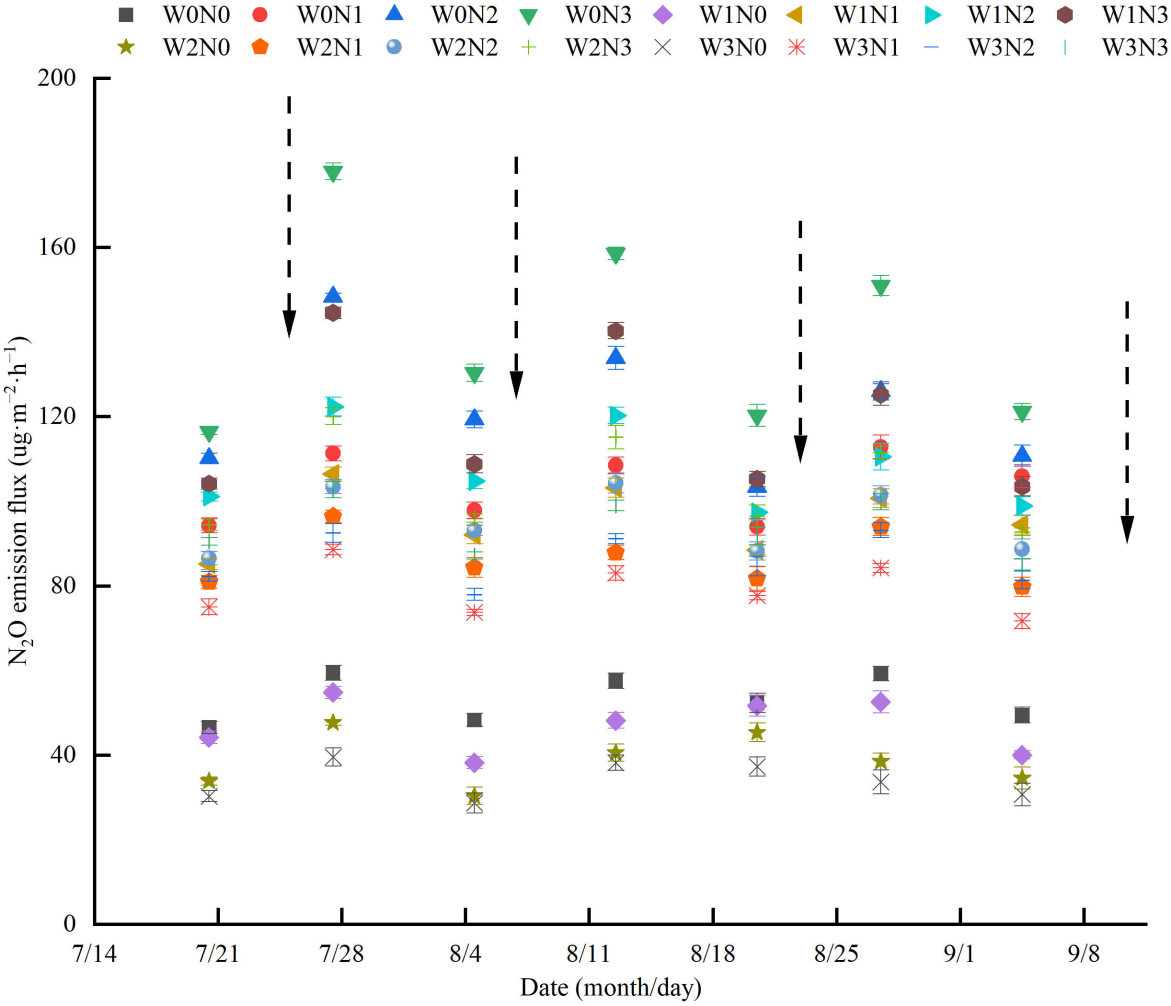
Effects of water and nitrogen regulation on soil N_2_O emission flux. Arrows indicate irrigation on the appropriate date. W0, W1, W2 and W3 refers to full irrigation (75%–85% θ_f_), slight water deficit (65%–75% θ_f_), moderate water deficit (55%–65% θ_f_) and severe water deficit (45%–55% θ_f_), respectively. N0, N1, N2 and N3 refers to the nitrogen application level is 0 kg·ha^−1^, 150 kg·ha^−1^, 300 kg·ha^−1^ and 450 kg·ha^−1^, respectively.

During the wolfberry harvest period, the soil N_2_O emission flux under different water and nitrogen treatments ranged from 28.68–177.91 ug·m^−2^·h^−1^. The pattern of change was consistent, with a peak occurring after irrigation, followed by a gradual decrease afterwards (**Figure 6**). In each period, under the same irrigation level, the soil N_2_O emission fluxes of N0, N1, and N2 decreased by 50.93%–68.38%, 8.77%–37.46%, and 2.95%–16.65% compared with N3. Under the same nitrogen application level, the soil N_2_O emission fluxes of W1, W2, and W3 decreased by 1.34%–20.89%, 13.00%–37.16%, and 17.34%–43.22% compared with W0. Among all treatments, W0N3 exhibited the highest soil N_2_O emission flux (116.39–177.91 ug·m^−2^·h^−1^), whereas W3N0 showed the lowest soil N_2_O emission flux (28.68–39.55 ug·m^−2^·h^−1^).

**Figure 7:**
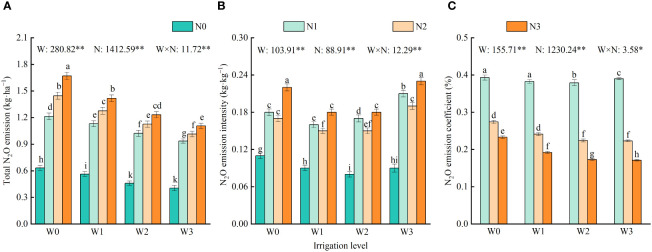
Effects of water and nitrogen regulation on total N_2_O emission, emission intensity and emission coefficient of soil. Different lowercase letters indicate significant differences between treatments (*P <* 0.05). **(A–C)** represents the total N_2_O emission, N_2_O emission intensity and N_2_O emission coefficient, respectively. W and N refer to irrigation and nitrogen application levels, respectively; N × W refers to interaction effect between the two. ** indicates an extremely significant difference (*P <* 0.01); * indicates a significant difference (*P* < 0.05). W0, W1, W2 and W3 refers to full irrigation (75%–85% θ_f_), slight water deficit (65%–75% θ_f_), moderate water deficit (55%–65% θ_f_) and severe water deficit (45%–55% θ_f_), respectively. N0, N1, N2 and N3 refers to the nitrogen application level is 0 kg·ha^−1^, 150 kg·ha^−1^, 300 kg·ha^−1^ and 450 kg·ha^−1^, respectively.

Irrigation, nitrogen application, and their interaction significantly affected the total amount, emission intensity, and emission coefficient of soil N_2_O (**Figure 7**). Overall, the total N_2_O emissions showed an increasing trend with increasing irrigation and nitrogen application. Compared with N0, the total N_2_O emissions from N1, N2, and N3 were increased by an average of 109.09%, 136.33%, and 163.73%, respectively. Compared with W0, the total N_2_O emissions from W1, W2, and W3 were reduced by an average of 11.60%, 22.63%, and 30.25%, respectively. Among all treatments, the total N_2_O emission of W0N3 was the highest (1.67 kg·ha^−1^), which was 0.22–1.27 kg·ha^−1^ higher than the other treatments.

Under the same irrigation level, N_2_O emission intensity followed the order N3 > N1 > N2 > N0. The emission intensities of N0, N1, and N2 were significantly reduced by 50.00%–60.87%, 5.56% –18.18%, and 16.67%–22.73%, respectively, compared with N3. The N_2_O emission coefficient decreased significantly with increasing nitrogen application. Specifically, N3 decreased by 50.19% and 20.05% compared with N1 and N2, respectively. Under the same level of nitrogen application, the intensity of N_2_O emissions initially decreased and then increased with increasing irrigation amount. The emission intensity of W0 decreased by significantly -22.22%–14.29% compared with that of W3. At the N1 level, the N_2_O emission coefficient initially decreased and then increased with increasing irrigation amount. At the N2 and N3 levels, the emission coefficients of N_2_O were W0 > W1 > W2 > W3 with the increase of irrigation amount, and the emission coefficients of W1, W2, and W3 decreased by 14.59%, 21.71%, and 22.25%, respectively, compared with W0. Among all treatments, the emission intensity and emission coefficient of N_2_O reached the maximum in W3N3 (0.23 kg·kg^−1^) and W0N1 (0.39%). These values increased by 4.56%–187.50%, and 0.74%–129.62%, respectively, compared with the other treatments.

#### 3.3.2 Relationship between soil N_2_O emission flux and environmental factors

**Figure 8:**
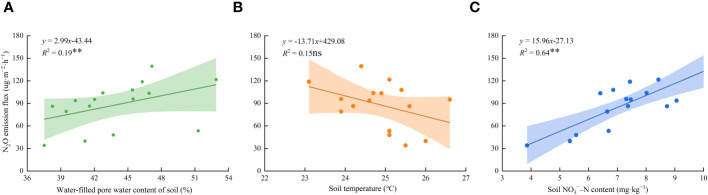
The relationship between soil N_2_O emission flux and environmental factors under different water and nitrogen regulation. The data in the figure are average values. **(A–C)** represents the water-filled pore water content of soil, soil temperature and soil NO_3_^−^–N content, respectively. Dots in the figure represent N_2_O emission fluxes; the linear line represents the linear fitting curve of N_2_O emission flux. The shaded band represents the 95% confidence band of the N_2_O emission flux.

According to the relationship between soil N_2_O emission flux and environmental factors under varying water and nitrogen conditions (**Figure 8**), soil N_2_O emission flux showed a positive correlation with the water-filled pore water content of the soil and NO_3_^−^–N content. The determination coefficients reached 0.19 and 0.64, respectively. However, the soil N_2_O emission flux decreased linearly as soil temperature increased, and there was no significant correlation between the two.

### 3.4 Comprehensive evaluation based on CRITIC-entropy weights-TOPSIS model

**Figure 9:**
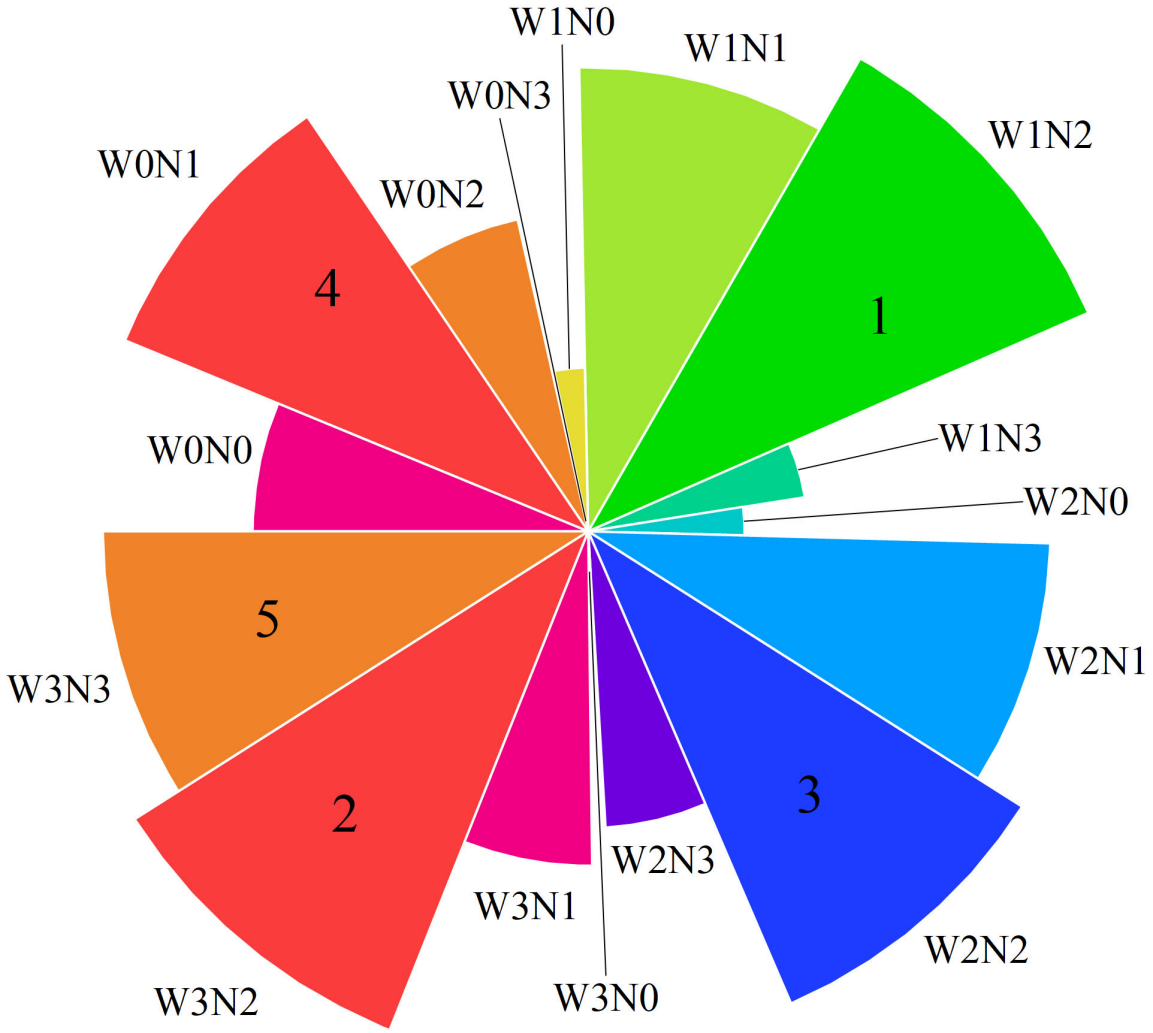
Comprehensive evaluation scores of different water and nitrogen regulation. The numbers 1, 2, 3, 4, and 5 in the figure represent W1N2, W3N2, W2N2, W0N1, and W3N3, respectively.

The total nitrogen content and total nitrogen uptake of wolfberry plants, soil NO_3_^−^–N accumulation, soil temperature, water-filled pore water content of the soil, N_2_O emission flux, and other indicators were consistently analyzed (**Table 3**). The weights of the wolfberry indices based on the CRITIC-entropy weight method were as follows: F > N_ua_ > NR_a_ > N_ia_ > f > GHGI > EF (**Table 4**). According to the comprehensive evaluation results of the CRITIC-entropy weights-TOPSIS model (**Figure 9**), the W1N2 treatment ranking was the best, followed by the W3N2 treatment. These results indicate that when a mild water deficit (W1, 65%–75% θ_f_) was coupled with medium nitrogen application (N2, 300 kg·ha^−1^), the nitrogen uptake of wolfberry plants was higher, and the accumulation of NO_3_^−^–N and N_2_O emission in the soil was lower.

**Table 3:** Consistency of TOPSIS indicator types.

Treatment	N_qa_	N_ua_	NR_a_	F	f	GHGI	EF
W0N0	0.096	0.013	5.280	0.019	1.581	9.431	
W0N1	0.091	0.009	84.724	0.010	0.824	5.662	2.581
W0N2	0.083	0.007	53.112	0.008	0.692	5.723	3.688
W0N3	0.088	0.008	0.000	0.007	0.599	4.466	4.338
W1N0	0.092	0.012	26.388	0.021	1.781	11.095	
W1N1	0.087	0.008	77.596	0.010	0.884	6.361	2.634
W1N2	0.078	0.006	95.676	0.009	0.783	6.896	4.196
W1N3	0.083	0.007	36.256	0.008	0.705	5.569	5.255
W2N0	0.102	0.014	24.868	0.025	2.178	12.422	
W2N1	0.096	0.010	77.384	0.012	0.979	5.981	2.665
W2N2	0.087	0.008	86.456	0.011	0.888	6.571	4.499
W2N3	0.091	0.009	49.184	0.010	0.811	5.695	5.819
W3N0	0.110	0.019	0.000	0.029	2.472	11.350	
W3N1	0.100	0.012	55.696	0.013	1.069	4.793	2.569
W3N2	0.091	0.010	94.908	0.012	0.986	5.140	4.473
W3N3	0.095	0.011	81.948	0.011	0.904	4.323	5.876

N_qa_, N_ua_ and NR_a_ represent the total nitrogen content and nitrogen uptake of each organ of the wolfberry plant, and the average value of NO_3_^−^–N accumulation in soil at different horizontal distances (30 cm, 60 cm, 90 cm, 120 cm and 150 cm), respectively. W0, W1, W2 and W3 refers to full irrigation (75%–85% θ_f_), slight water deficit (65%–75% θ_f_), moderate water deficit (55%–65% θ_f_) and severe water deficit (45%–55% θ_f_), respectively. N0, N1, N2 and N3 refers to the nitrogen application level is 0 kg·ha^−1^, 150 kg·ha^−1^, 300 kg·ha^−1^ and 450 kg·ha^−1^, respectively.

**Table 4:** Weights of each index based on CRITIC-entropy weight method.

Index	N_qa_	N_ua_	NR_a_	F	f	GHGI	EF
Information entropy (*E_j_*)	0.999	0.987	0.994	0.978	0.977	0.984	0.879
Information utility value (*C_j_*)	1.944	30.210	26.739	31.406	1.342	1.031	0.967
Weight (*W_j_,* %)	0.917	14.251	12.292	14.816	0.633	0.487	0.456

## 4 Discussion

### 4.1 Distribution and accumulation of soil NO_3_^−^–N under different water and nitrogen regulation

Soil NO_3_^−^–N is an important component of soil soluble nitrogen and serves as the primary mineral nitrogen source directly absorbed by crops. It is commonly used to assess the nitrogen supply capacity of the soil (Cui et al., 2013). This study revealed that the soil NO_3_^−^–N content in the 0–100 cm soil layer of each treatment decreased during the harvest period initially decreased, then increased, and finally decreased with increasing soil depth. Additionally, it decreased with increasing horizontal distance from the wolfberry plants. The soil NO_3_^−^–N content at 30 cm and 60 cm from the wolfberry followed the order of W0 > W2 > W1 and W3. This can be attributed to two factors: Firstly, the negative charge of the NO_3_^−^ ions causes repulsion with the negatively charged soil colloidal particles, leading to the downward movement of NO_3_^−^–N through leaching and infiltration, thereby affecting the vertical redistribution of NO_3_^−^–N. Secondly, water migration and root absorption affect the vertical and horizontal redistribution process of NO_3_^−^–N, resulting in intensified NO_3_^−^–N leaching near the wet body in the drip irrigation zone and the phenomenon of “enrichment” near the roots (Vijayalakshmi et al., 2013). Furthermore, this study revealed a positive correlation between soil NO_3_^−^–N content and the amount of nitrogen applied. The NO_3_^−^–N content increased with the amount of irrigation and nitrogen application, and the peak value of NO_3_^−^–N increased and gradually moved downward. This finding was similar to that reported by Gu et al. (2018) for winter oilseed rape in the Guanzhong area of Shaanxi Province. This result indicated that urea applied to the soil through irrigation rapidly hydrolyzes into NH_4_^+^–N and is oxidized to NO_3_^−^–N under the action of ammonia oxidizing microorganisms. However, NO_3_^−^–N is highly soluble in water and tends to accumulate at a specific depth within soil pores (Wu et al., 2022b).

Crops absorb a portion of NO_3_^−^–N during their growth, whereas unabsorbed NO_3_^−^–N accumulates in the soil, thereby increasing the potential risk of groundwater pollution. In this study, the accumulation of soil NO_3_^−^–N in the 0–100 cm soil layer decreased as the horizontal distance increased under different water and nitrogen treatments (except for the N0 nitrogen application level). The average soil NO_3_^−^–N accumulation in the 0–100 cm soil layer at each horizontal distance followed the order of W0 > W1 > W2 > W3 (except for the N2 nitrogen application level), and N3 > N2 > N1 > N0. Both water and nitrogen affected the accumulation of NO_3_^−^–N in the soil, with nitrogen application having a greater influence on soil NO_3_^−^–N accumulation than the irrigation amount (**Table 3**). This contradicts the findings of Wang et al. (2008) in Shandong Province, which indicated that both irrigation and nitrogen application significantly affected NO_3_^−^–N accumulation in the 0–100 cm soil layer of wheat, and the contribution of irrigation to NO_3_^−^–N accumulation was greater than that of nitrogen application. This may be attributed to the more developed root system of wolfberry than that of wheat. Additionally, the soil resource endowment characteristics in the Yellow River irrigation region of Gansu Province may not fully support the growth and development of wolfberry, leading to the increased reliance on exogenous nutrients to maintain normal physiological growth activities of the plants (Dou et al., 2021). At the same time, this study concluded that the single factor of water and nitrogen significantly affected the accumulation of soil NO_3_^−^–N. The interaction effect of the two factors only had a significant impact on NO_3_^−^–N accumulation in the soil 30 cm away from the horizontal distance of the wolfberry (**Table 2**).This may be related to the root distribution of wolfberry plants, and multiple irrigations may have caused downward leaching of nitrogen accumulation. Nitrogen accumulation tends to decrease away from the drip irrigation belt, making wolfberry roots closer to the belt more sensitive to water and nitrogen responses (Iranzi et al., 2023).

### 4.2 Nitrogen allocation and uptake of wolfberry plants under different water and nitrogen regulation

A reasonable water and nitrogen supply pattern is beneficial for increasing nitrogen uptake by plants (Dai and Zhang, 2020). The results of this study revealed that the total nitrogen content of each organ of the wolfberry at the time of harvest followed the order leaves > roots and stems > fruits. The highest values were observed under the W1N2 treatment, with the nitrogen content reaching 3.25% in the roots, 3.30% in the stems, 3.91% in the leaves, and 2.42% in the fruits. This suggests that the appropriate water-nitrogen combination has a synergistic effect on nitrogen allocation and uptake in crops (Qin et al., 2021). In addition, this study found that the total nitrogen content of wolfberry leaves and fruits initially increased and then decreased with increasing irrigation and nitrogen application. This contradicts the findings of Zhen and Zhen (2006) and Qin et al. (2017) who concluded that the nitrogen content in wheat leaves and fruits is regulated by exogenous nitrogen and increases with increasing nitrogen application. The difference in absorption capacity between wolfberry and wheat could be attributed to the more developed roots and stronger absorption capacity of wolfberry. Additionally, nitrogen application in this experiment reached the absorption threshold of wolfberry (medium nitrogen application of 300 kg·ha^−1^). With an increasing in nitrogen application (high nitrogen application of 450 kg·ha^−1^), the antagonism between the absorbed elements and ions of wolfberry plants was enhanced. This leads to extravagant nitrogen absorption, by the plants, ultimately resulting in a decrease in the nitrogen content of crops (Filipović et al., 2016).

Irrigation and nitrogen application can affect the accumulation of plant nutrients by improving soil water and fertilizer conditions (Liu et al., 2007). In this study, it was concluded that the nitrogen uptake of wolfberry showed the following trend: N2 > N3 > N1 > N0. Specifically, N2 showed significant increases of 85.32%, 26.80%, and 15.39% compared with N0, N1, and N3, respectively. These finding are consistent with those of a study on cotton in the Xinjiang region conducted by Zhang et al. (2021). This may be due to the excessive application of nitrogen, which can lead to an imbalance in plant nutrient uptake, reduce plant nitrogen uptake and accumulation, and cause loss of nitrogen resources. However, appropriate nitrogen application can increase the levels of inorganic nitrogen, such as NO_3_^−^–N and NH_4_^+^–N in the soil of the root zone, and facilitate nitrogen uptake and accumulation in plants (Gu et al., 2018). The study concluded that the nitrogen uptake of wolfberry initially increased and then decreased with increasing irrigation amount. The total nitrogen uptake of W0 was significantly lower than that of W1 by 11.31%–12.50%. However, the accumulation of NO_3_^−^–N showed a fluctuating trend and W0 significantly increased by 7.54%–46.61% compared with W3. This suggests that a reasonable increase in irrigation can enhance crop nitrogen uptake, whereas excessive irrigation can lead to the “dilution effect” of crop nitrogen and exacerbate soil nitrogen loss, including the leaching of NO_3_^−^–N and NH_4_^+^–N, N_2_O emission, and NH_4_ volatilization (Ma et al., 2023). In addition, this study also revealed that the nitrogen uptake of wolfberry was the highest in the fruits, followed by the stems, roots, and leaves (except for the W3 treatment). Among these, the nitrogen absorption rate of wolfberry fruit (63.56 kg·ha^−1^) was the highest, and the total nitrogen absorption rate of wolfberry plants (160.45 kg·ha^−1^) was the highest under W1N2 treatment. It can be observed that using the appropriate water and nitrogen management strategy can produce a synergistic effect on water and nitrogen, enhancing the activity of nitrogen metabolism enzymes and the uptake of nitrogen by plants (Sun et al., 2009).

### 4.3 Soil N_2_O emission and its influencing factors under different water and nitrogen regulation

The application of irrigation and nitrogen not only affects the distribution and accumulation of NO_3_^−^–N in the soil and distribution and the absorption of nitrogen by plants, but also affects soil N_2_O emissions. The fluxes of N_2_O emission and total emissions during the harvest period of wolfberry were 28.68–177.91 ug·m^−2^·h^−1^ and 0.40–1.67 kg·ha^−1^, respectively. Moreover, three peaks of N_2_O emission flux (177.91 ug·m^−2^·h^−1^, 158.55 ug·m^−2^·h^−1^, and 150.93 ug·m^−2^·h^−1^) occurred after irrigation. This suggests that irrigation can increase soil microbial abundance and soil enzyme activity, leading to an increased mineralization rate of soil organic matter, higher soil nitrogen content, and consequently, greater release of N_2_O (Li et al., 2020). In line with Du et al. (2018), this study also found that the N_2_O emission flux from fully irrigated fields increased by an average of 7.97%–53.69% compared with deficit irrigation. This may be because higher soil moisture reduces soil porosity and soil O_2_ diffusion capacity, enhances soil denitrification, and promotes N_2_O emissions. The addition of exogenous nitrogen increases the substrate concentration for soil nitrification and denitrification, leading to enhanced N_2_O emissions (Stehfest and Bouwman, 2019). The results of this study revealed a significant positive correlation between total N_2_O emissions and nitrogen application (**Figure 7A**). Additionally, the total N_2_O emissions at 300 kg·ha^−1^ were 13.03% higher than those at 150 kg·ha^−1^. Similar results were also found by Zheng et al. (2021) in their study on wheat in Northwest China. This study also concluded that the interaction between water and nitrogen had a significant impact on the N_2_O emission intensity. The N_2_O emission intensity of the W1N2 treatment was significantly reduced by 31.8% and 11.76% compared with the W0N3 and W2N1 treatments (**Figure 7B**). These results indicate that the intensity of N_2_O emissions can be effectively reduced by appropriately reducing the amount of irrigation water and nitrogen application (Zhang et al., 2015). Previous studies found that the N_2_O emission coefficients of vegetable plots under different water and nitrogen supplies ranged from 1.09%–1.63% under different water and nitrogen conditions. Additionally, the N_2_O emission coefficients of orchards initially decreased and then increased with increasing nitrogen application (Cao et al., 2006; Wang et al., 2019). In this study, the N_2_O emission coefficient (0.17%–0.39%) for all treatments was generally low and decreased with increasing nitrogen application. This reason may be because the irrigation frequent during the growth period of wolfberry lower than that in vegetable fields, and frequent irrigation can lead to frequent alternations of dry and wet soil, promoting N_2_O emissions.

N_2_O emission fluxes are influenced by various factors, such as WFPS, soil temperature, and soil NO_3_^−^–N content. It has been found that soil wet-dry cycles promote the emission of N_2_O by stimulating nitrification and denitrification (Peyron et al., 2016). The emission flux of N_2_O is exponentially correlated with WFPS (Du et al., 2018). WFPS is maintained at 45%–75% when the N_2_O emission rate is at its maximum, during which soil nitrification-denitrification produces the same proportion of N_2_O (Shelton et al., 2000). This study demonstrated that soil WFPS in the 0–15 cm soil layer ranged from 37.53%–52.90% under varying water and nitrogen regulations. Additionally, a significant positive linear correlation was observed between N_2_O emission flux and WFPS (**Figure 8A**). This indicates that when WFPS was low, N_2_O emissions were mainly from the nitrification reaction, and WFPS gradually increased with increasing irrigation levels. When the WFPS exceeded a certain threshold, the denitrification rate gradually accelerated and contributed to N_2_O emissions along with the nitrification reaction. Soil temperature is an important factor that influences plant root respiration and soil microbial activity. Chen et al. (2018) found an exponential positive correlation between N_2_O emission flux and soil temperature in their study of greenhouse tomato in northwest China. This finding contradicted the conclusion of the present study, which found a linear and negative correlation between N_2_O emission flux and soil temperature (*R* ^2 =^ 0.15ns). The variation in soil temperature (23.1°C–26.6°C) during the wolfberry harvest period suggested a decrease in soil nitrogen mineralization and soil microorganism respiration. Simultaneously, the soil NO_3_^−^–N content can not only promote the denitrification rate but also inhibit the reduction of N_2_O to N_2_. In this study, it was found that soil NO_3_^−^–N increased with increasing nitrogen application (**Figure 3**; **Table 2**). Additionally, N_2_O emission flux showed a significant and linear positive correlation with soil NO_3_^−^–N content (*R* ^2^ = 0.64**). This result further demonstrated that when the nitrogen application rate exceeds the nitrogen requirement of the plant, excess nitrogen remains in the soil and is eventually lost in the form of N_2_O. Therefore, water and nitrogen inputs should be properly controlled to minimize N_2_O emissions during agricultural production. In addition to WFPS, soil temperature, and soil NO_3_^−^–N content, the intermediate products of nitrification-denitrification, N_2_, and NH_4_, are also important factors that affect soil N_2_O emissions. Subsequent monitoring of N_2_ and NH_4_ should be conducted to further investigate the emission characteristics of soil N_2_O under different water nitrogen regulations.

## 5 Conclusions

Soil NO_3_^−^–N content exhibited a leaching trend with an increase in irrigation amount and an increasing trend with an increase in nitrogen application rate. Soil NO_3_^−^–N accumulation (90.13–185.91 kg·ha^−1^) gradually decreased with the increase in horizontal distance and increased with the increase in irrigation and nitrogen application. The total nitrogen content and uptake in all organs (roots, stems, leaves and fruits) exhibited threshold values in response to water and nitrogen, reaching their maximum under the W1N2 treatment. The maximum values were 3.25% and 27.82 kg·ha^−1^, 3.30% and 57.19 kg·ha^−1^, 3.91% and 11.88 kg·ha^−1^, 2.42% and 63.56 kg·ha^−1^, respectively. The flux of N_2_O emission ranged from 28.68–177.91 ug·m^−2^·h^−1^, with total emissions ranged from 0.40–1.67 kg·ha^−1^. The emission intensity varied from 0.08–0.23 kg·kg^−1^ and the emission coefficient ranged from 0.17%–0.39%. These values exhibited an increasing trend with increasing irrigation. The total emission, emission intensity and emission coefficient of N_2_O reached their highest values in the W0N3 treatment (1.67 kg·ha^−1^), W3N3 treatment (0.23 kg·kg^−1^) and W0N1 treatment (0.39%) treatments, respectively. Based on the comprehensive evaluation of the CRITIC-entropy weights-TOPSIS model, it was concluded that a slight water deficit (65%–75% θ_f_) coupled with a nitrogen application rate (300 kg·ha^−1^) is an effective water and nitrogen control model to conserve water and reduce nitrogen in the production of wolfberry in the Yellow River irrigation region of Gansu Province.

## Data availability statement

The raw data supporting the conclusions of this article will be made available by the authors, without undue reservation

## Author contributions

RT: Conceptualization, Data curation, Formal analysis, Investigation, Methodology, Writing – original draft. JW: Conceptualization, Project administration, Supervision, Writing – review & editing. MY: Data curation, Formal analysis, Writing – review & editing. YM: Funding acquisition, Writing – review & editing. QJ: Funding acquisition, Writing – review & editing. YK: Funding acquisition, Writing – review & editing, Project administration. GQ: Formal analysis, Writing – review & editing. YG: Writing – review & editing, Investigation. YJ: Writing – review & editing, Methodology. HL: Writing – review & editing, Project administration. FX: Investigation, Writing – review & editing.
